# Exploring the Mode of Entrepreneurship Education Based on the Legal-Business Compound Competency in China

**DOI:** 10.3389/fpsyg.2019.01055

**Published:** 2019-05-09

**Authors:** Ting Wang

**Affiliations:** School of Business, China University of Political Science and Law, Beijing, China

**Keywords:** entrepreneurship education, legal-business compound competency, law–business interdisciplinary talents, competency model, behavioral event interview

## Abstract

A perfect legal guarantee can ensure China’s high-quality socio-economic development. At present, in terms of China’s entrepreneurship education (EE), it is necessary to strengthen entrepreneurs’ legal consciousness and respect for rules. The research establishes a model for legal-business compound competency (LBCC). It is a pioneering EE mode adapted to characteristics of China’s market transition to cultivate interdisciplinary talents who excel at management and administration but also have a command of laws and regulations in EE. By utilizing behavioral event interview (BEI) and Delphi methods, factors affecting LBCC were summarized. Moreover, a questionnaire-based inquiry was conducted using graduates who received law–business interdisciplinary entrepreneurship education (LBIEE) as subjects to collecting data to evaluate the cultivation effect of the EE mode. In the study, a model for LBCC was established from the three perspectives including knowledge, skill, and attitude. Additionally, cultivating competency of law–business interdisciplinary talents (LBITs) shows a significantly positive influence on compensation level and job satisfaction among graduates who have received the EE. The core task of LBIEE is to improve compound competency of students in legal-business to enable students to show entrepreneurial spirit with legal-business intelligence. It is considered an innovation in a mode of education adapted to the transition and development of China’s market economy.

## Introduction

Entrepreneurship education (EE) pursues the development of student competency to grasp commercial opportunity (Daniela et al., 2016) and adapt to complex business environments (Ho et al., 2014; Rauch and Hulsink, 2015). Whether and how competency is cultivated through EE is proposed by Gorman et al. (1997). It is suggested that competency can be favorably cultivated via EE (Kuratko, 2005) while related meta-analysis also shows that EE is, on the whole, effective (Martin et al., 2013; Bae et al., 2014); however, super-fine multidisciplinary settings in educational contexts leads students passively receiving separate disciplinary cultures (Gu, 2011). As a result, it is hard for students to develop a comprehensive competency in solving realistic problems in society (Gu, 2011). In the new era, with rapidly changing technologies and an ever-changing market environment, entrepreneurs need to face significant uncertainty. Therefore, they have to retain multidisciplinary knowledge and skills and show strong adaptability. In contrast, the essence of interdisciplinary talents lies in combination of knowledge, skill, and quality and therefore interdisciplinary talents are essential in the new era (Su and Ma, 2011). Cultivating interdisciplinary talents is an important direction for development research and practice in EE (Gary, 2005; Daniela et al., 2016; Virginia and Carlos, 2018).

The cultivation of interdisciplinary entrepreneurial talents will meet the demand of a society for comprehensive, application-oriented, and innovative talents to promote the development of society, the economy, and scientific technology (Wang and An, 2015; Karen and Dawn, 2016). The mode of innovation of inter-disciplinary talent cultivation in EE is constantly acknowledged and strengthened. Many colleges start to integrate business education with other disciplines, expecting to cultivate inter-disciplinary entrepreneurial talents. In the United States, there is no doubt that JD (Juris Doctor) or MBA (Master of Business Administration) are the most characteristic and attractive co-training projects (Chen and Qiu, 1996). Law–business interdisciplinary entrepreneurship education (LBIEE) underwent its first 5-year test period at the University of Virginia: this proved that inter-disciplinary education in law and management was feasible (John, 1982). Afterward, Harvard Business School and Northwestern University (United States) also expanded similar EE projects to cultivate LBITs (Lynn and Justin, 2005). Through JD/MBA education project, Harvard University aims to train students to master rigorous and centralized classroom knowledge, acquire practical expertise in law and management, and deepen their understanding of legal and business principles in their future career (Wang, 2017). However, Northwestern University’s JD/MBA project pays more attention to practice, thus providing students with many practical learning opportunities to help them deal with many legal and business cross-cutting incidents (Wang, 2017).

A series of research achievements show that the cultivation characteristics of EE are influenced by their environment (Walter and Dohse, 2012; De Clercq et al., 2013), institution (Sascha and Jörn, 2016), and national background (Shepherd, 2011; Panagiotis, 2012). With the further development of legalization processes in China’s economy, entrepreneurs will inflict a destructive blow to a start-up if they make a decision violating laws and business ethics (Baron et al., 2015). China’s economic development during the transition period requires a great number of interdisciplinary talents who not only have a command of management but also show legal awareness (Wang, 2017). Therefore, in China’s EE, it is necessary to strengthen the learning of law-based knowledge and cultivation of rule consciousness (Chai and Sun, 2012; Qu, 2015). In particular, with the internationalization of Chinese Enterprises, mastering the legal operating norms of overseas markets have been one of necessary attributes of entrepreneurial talents (Sun, 2018).

In 2010, Business School of China University of Political Science and Law (CUPL) started to explore the innovation of the law–business EE mode based on the construction of a pilot site for cultivating LBITs in quality engineering by the Ministry of Education of the People’s Republic of China (Sun et al., 2012). China’s LBIEE has been a new field which is under development in many universities (Huang et al., 2012; Zhao, 2015). A market economy means an economy ruled by law, so law is naturally integrated with business, which is an innate demand of entrepreneurial management (Wu, 2012). The inclusion of the legal and ethical components in business education programs can provide the tools and processes necessary for executive decision making (Ostapski et al., 1996). The LBIEE mode emphasizes the integration of law and business elements (Li, 2012) to remove boundaries between disciplines (Andrew et al., 1996). In particular, it effectively solves problems arising during the transition of China’s market economy (Xie, 2017; Wang, 2018) and improves the law–business interdisciplinary application ability of students in commercial activities by applying interdisciplinary education methods (Wang, 2017). Students that have a better understanding of the legal issues involved with invention and entrepreneurship can have more successful careers and better contribute to society (Borns, 2002).

According to the psychologically educational theory, the competency is the integration of knowledge, skill and attitude (KSA) which affect performance and hence the success of the individual (McClelland, 1973; Baartman and Bruijn, 2011). This research explores a model for legal-business compound competency (LBCC) established from these three perspectives including knowledge, skill, and attitude through behavioral event interview (BEI) which is the general method in the research of competency exploration. Then, we evaluate the influence of the LBIEE on the graduates’ compensation level and job satisfaction by the survey data from CUPL. The purpose of this research is to improve the LBIEE mode by exploring characteristics of LBCC.

## Materials and Methods

For exploring the model of LBCC, the method of BEI is firstly executed to extract the competency factors of LBCC. Then, through the Delphi method, the experts involved to the LBIEE project are invited to score the importance degree of these factors for making the extracted factors more complete and effective. According to the process of BEI and Delphi methods, some of the most important characteristics of competency of LBITs can be ranked in qualitative analysis. Moreover, the scales for competency were formed in the survey questionnaire to collect the data for establishing a LBCC model in three perspectives of knowledge, skill, and attitude by extraction method of principal component analysis and quantitatively evaluating the influence of LBIEE project on the graduates.

### Behavioral Event Interview

The method of BEI is an open exploration technology based on behavior review (McClelland, 1973; Spencer and Spencer, 1993; Larson, 2001). It is implemented according to the following process: interviewees narrate two or three successful and failed typical cases in their working lives and explain the whole process (including situation, participants, actions, thinking, and result) of events in detail. In the interview process, it is necessary to conduct effective guidance, and record the process. Finally, professionals make a summary, analysis, and code their findings to reveal core qualities in the staff interviewed (Adrian et al., 2014).

Through expert recommendation, 54 people with outstanding managerial ability in legal-business were interviewed. By utilizing BEI, 190 legal–business management cases were extracted in which there were 94 successful and 96 failed cases, respectively. On this basis, competency factors were extracted and coded from the cases to form a competency dictionary relating to LBCC. Moreover, frequencies of various competency factors in cases were calculated (Table 1).

**Table 1:** Dictionary of competency factors in legal-business and statistical results (frequencies).

Factor of competency	Sub-dimension	Name and frequency
Knowledge	Knowledge in management	Knowledge concerning managerial economics (11), strategic management (36), leadership (7), financial management (13), marketing (3), contract management (12), human resource management (HRM) (45), organizational behavior (6), quality management (3), supply chain management (3), international operation (4)
	Knowledge in law	Knowledge concerning contract law (100), labor law (34), intellectual property law (12), trademark law (5), competition law (4), overseas legal risk of enterprises (4), commercial law (24), securities law (5), tax law (3), procedural law (2)
Skill	Basic skill	Negotiation ability (32), ability of interpersonal skill (3), communication ability (52), ability of analysis and identification (29), executive capacity (23), flexible application ability (18), learning ability (8), innovation capacity (6), self-discipline ability (12), position competency (14), stress resistance (3), ability to understand policy (15)
	Managerial skill	Team-based organization ability (11), strategic decision-making ability (13), risk management capacity (117), resource integration capability (15), ability of crisis management (37), cross-cultural management ability (5)
Attitude		Legal consciousness (135), rule consciousness (37), consciousness of rights safeguarding (50), sense of responsibility (17), overall viewpoint (33), rationality and calmness (43), carefulness (44), dialectical thinking (9), aggressiveness (8), objectivity and fairness (4), win-win cooperation (11), caution (42), honesty and trustworthiness (9), tenacity and determination (1)


*Notes: Figure in the parentheses refers to the frequency of the competency factor in cases based on BEI.*

### Delphi Method

The Delphi method, also called the expert inquiry investigation method, is, in essence, an anonymous feedback inquiry method. The method can predict investigated problems through comprehensive analysis, which is implemented based on the following process: according to a rule-based procedure, anonymous experts are consulted for opinions or judgment of some predicted problems; According to the results of several investigations, and by virtue of knowledge and experience of experts, arrangement, statistics, and calculations are undertaken, and conclusions reached (Jon et al., 2017).

In the study, 27 teachers involved in this LBIEE project were invited to take part in the expert inquiry. The result obtained through the BEI was sent to various experts by e-mail. In this way, the experts can score the degree of importance of various factors concerning LBCC in Table 1 and also compensate for the dictionary of competency factors. After being subjected to two rounds of feedback, and supplemented by use of the Delphi method, the variance, standard deviation, etc., of competency factors in the second round were lower than those first obtained, and the dot pitch of upper and lower quartiles also slightly reduced. It indicated that experts’ feedback was more concentrated after two rounds of feedback and supplementation through the Delphi method: the mean of scores, deduced by experts for various factors of competency of LBITs in the dictionary, have high reliability (Petra, 2016; Stefan et al., 2017). Moreover, according to expert opinions obtained based on the Delphi method, three competency factors (including knowledge of business ethics, accountancy knowledge, and knowledge of corporate governance) are added into “knowledge in management.” Moreover, a competency factor (comprehensive thinking ability) is supplemented for “basic skill.” The “attitude” also contains team awareness and achievement motivation; therefore, the dictionary of factors of competency of LBITs is rendered more complete.

According to statistical data pertaining to scores by experts, the top 12 ranked competency factors in terms of scores are listed in Table 2, and mainly concentrate on attitude and skill factors. This implies that the skill factors in an iceberg competency model (McClelland, 1973) and attitude factors under ice surface are of great importance to EE. The factor “honesty and trustworthiness” returned the highest score, which is the most important characteristic of competency of LBITs and the factor which most needs cultivating during LBIEE.

**Table 2:** Factors of LBCC ranked in terms of scores by experts based on the Delphi method.

Rank	Name of competency factors	Mean	Standard deviation	Variance	Dimension of competency
1	Honesty and trustworthiness	4.67	0.483	0.233	Attitude
2	Communication ability	4.57	0.598	0.357	Skill
3	Learning ability	4.57	0.507	0.257	Skill
4	Team-based organization capacity	4.57	0.598	0.357	Skill
5	Strategic decision-making capacity	4.57	0.598	0.357	Skill
6	Legal consciousness	4.52	0.68	0.462	Attitude
7	Rule consciousness	4.52	0.68	0.462	Attitude
8	Sense of responsibility	4.52	0.68	0.462	Attitude
9	Overall viewpoint	4.52	0.602	0.362	Attitude
10	Innovation capacity	4.48	0.75	0.562	Skill
11	Knowledge of strategic management	4.48	0.68	0.462	Knowledge
12	Knowledge of contract law	4.43	0.87	0.757	Knowledge


### Data Collection Based on Questionnaire Survey

To reflect the quality of cultivation of LBITs through this EE project, some questionnaires were issued to graduates who received LBIEE. The contents of questionnaires involved basic information including the evaluation of research objects on LBIEE and working conditions after they received EE. Additionally, based on factors affecting LBCC formed by using BEI and the Delphi method, a 5-point Likert scale for competency including 24 knowledge factors, 19 skill factors, and 16 attitude factors was established and used to evaluate ability levels of research objects.

During questionnaire design, to avoid common method bias, the following methods were used: (1) interviewees anonymously fill in questionnaires; (2) interviewers promise confidentiality and require interviewees to answer questions as honestly as possible; (3) interviewers define and explain any ambiguous or specialized professional questions (Nederhof, 1985; Podsakoff et al., 2003).

The Business School of China University of Political Science and Law first proposed LBIEE in China and has attracted a diversified elite cadre of students for enterprise management. It can represent the cultivation of demand and characteristics of competency of a majority of LBITs to some extent. On the premise of it being both voluntary and anonymous, interviewers issued paper and electronic questionnaires to law–business interdisciplinary graduates in the university who received LBIEE. All of the 145 questionnaires were returned (an effective return rate of 100%). The numbers of male and female samples were 89 and 56, which accounted for 61.4 and 38.6% of the whole sample, respectively. It approximated to the overall male–female ratio of graduates in the university who participate in LBIEE. The research objects are mainly aged from 30 to 40 years old and they are engaged in diversified careers. Therefore, the structure of investigated samples was deemed reasonable.

### Test of Reliability and Validity of Questionnaires

Statistical analysis was conducted on the collected data. The values of Cronbach’s alpha of various factors of LBCC in a Likert scale are all larger than 0.9 and the total α value in the scale reaches 0.964 (Table 3). This indicated that the scale for competency applied in the study shows a high reliability (James, 1993). Moreover, the Kaiser-Meyer-Olkin (KMO) values of various variables are all larger than 0.8. According to the reference standard of KMO value, it can be seen that there is a strong correlation between variables. Therefore, KMO is favorably suitable for factor analysis. The corresponding probability (*p*) of observed values of statistical values obtained based on Bartlett’s test of sphericity is less than 0.0001, suggesting a significant difference, therefore, Bartlett’s test of sphericity is also suited to factor analysis (Table 4). Based on the aforementioned data, it can be seen that the scale for competency exhibits favorable validity (James, 1993).

**Table 3:** Test index for reliability of questionnaires.

Variable	Variable factor	Measured item	α value of various factors	Total α of the scale
LBCC	Knowledge	24	0.945	0.964
	Skill	19	0.934	
	Attitude	16	0.945	


**Table 4:** Indices for KMO and Bartlett’s test of sphericity of questionnaires.

Variable	Verified value for sample sufficiency based on KMO	Approximate chi-square value verified by using Bartlett’s test of sphericity	Degree of freedom	Sig.
LBCC	0.863	7282.257	1711	<0.0001


## Results

### Benefits From LBIEE Project

Data obtained through this investigation revealed that graduates think that they mainly benefit from the following aspects: systematically learning managerial knowledge, enhancing the consciousness of legal risk prevention, enriching interpersonal networks, expanding commercial opportunities, etc., through participation in this LBIEE project. The proportions of benefits in these four aspects are 73.8, 71.0, 64.1, and 60.7%, respectively (Table 5), showing that the EE project realizes the training objective of gaining improvements in legal-business contexts. With the progressive improvement of China’s market economy and the increase of the degree of internationalization therein, entrepreneurs need to be good at management but also proficient in mastering its rules. That is, they are required to have business intelligence and also be able to grasp and apply rules (Sun, 2018). Facing such a context, strengthening learning in law courses in EE is conducive to contributing to the success of graduates in innovation and when starting a business (Wang, 2018). The LBIEE project proposed by China University of Political Science and Law strengthens the competitiveness of graduates in complex business environments and also provides high-quality LBITs for China’s market economy in the transition period.

**Table 5:** Benefits from LBIEE project.

Benefits from learning	Number of samples	Proportion of total sample
Systematically learning managerial knowledge	107	73.8%
Strengthening consciousness of legal risk prevention	103	71.0%
Enriching interpersonal network	93	64.1%
Expanding business opportunity	88	60.7%
Broadening the horizon and renewing ideas	58	40.0%
Improving practical ability	29	20.0%
Others	5	3.5%


### Establishing a Model for LBCC

Competency was first proposed in research by an American psychologist McClelland (1973): by establishing an iceberg competency model, he suggested that personal qualities influencing work performance can be divided into knowledge, skill, self-cognition, quality, and motivation. Competency is the potential characteristics of a person, which enable them to attain excellent performance in their posts (Boyatzis and Royatzis, 1982), and it is the technology, ability, and personal characteristics required by an efficient or competent manager (Page et al., 1994). Overall, competency is defined as the requirement of a specific work for a person in knowledge, skill, and attitude (Spencer and Spencer, 1993). The LBCC model will be established based on these three aspects.

### Establishment of the Model for LBCC Based on Knowledge Factors

In terms of knowledge, according to the characteristics of LBIEE, 24 items from two major categories (knowledge in management and law) are extracted from the scale used for LBCC, as shown in Table 1. Through the SPSS software-based tests, the KMO value reflecting the overall validity of knowledge factors in the questionnaires was 0.888. This indicated that various knowledge factors in the questionnaires show many common factors and therefore the validity obtained through factor analysis is feasible. Factor analysis was conducted by applying principal component analysis (PCA) and varimax rotation was used to perform orthogonal rotation on the factor load matrix: four factors were thus acquired. The interpretation rate of the factors on the total variance was 67.174% and the result of factor analysis is shown in Table 6.

**Table 6:** Result of factor analysis on knowledge factors.

	Component			
	
	1	2	3	4
Labor law	0.846	0.102	0.163	-0.043
Contract law	0.759	0.264	0.203	0.091
Human resource management	0.686	-0.087	0.509	0.021
Procedural law	0.679	0.413	-0.150	0.199
Contract management	0.651	0.234	0.374	0.234
Intellectual property law	0.643	0.276	0.047	0.424
Trademark law	0.641	0.324	0.039	0.509
Competition law	0.549	0.469	0.140	0.505
Corporate governance	0.483	0.373	0.403	0.134
Business ethics	0.462	0.097	0.288	0.419
Accountancy	0.105	0.808	0.184	0.035
Securities law	0.261	0.774	0.075	0.201
Financial management	-0.024	0.720	0.445	0.078
Tax law	0.356	0.659	0.257	0.173
Business law	0.486	0.599	0.134	0.264
Overseas legal risk	0.343	0.562	0.165	0.442
Strategic management	0.140	0.195	0.747	0.269
Leadership	0.210	0.124	0.737	0.133
Marketing	0.076	0.412	0.659	0.227
Managerial economics	0.159	0.301	0.655	0.241
Organizational behavior	0.581	-0.039	0.590	0.147
Supply chain management	0.030	0.154	0.245	0.787
Quality management	0.214	0.063	0.244	0.701
International operation	0.068	0.325	0.441	0.522


*Extraction method, principal component analysis.*

**FIGURE 1:**
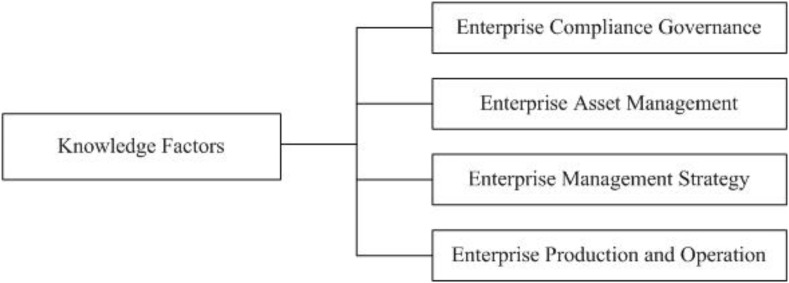
LBCC model based on knowledge factors.

According to the aforementioned factor analysis, four factors can be extracted from 24 knowledge factors. Among them, knowledge of labor law, contract law, HRM, procedural law, contract management, intellectual property law, trademark law, competition law, corporate governance, and business ethics show a high load in the first factor, which can be called knowledge of Enterprise Compliance Governance; knowledge of accountant, securities law, financial management, tax law, business law, and overseas legal risk of enterprises exhibit a high load in the second factor, which can be named knowledge of Enterprise Asset Management; knowledge of strategic management, leadership, marketing, managerial economics, and organizational behavior have a high load in the third factor, which is called knowledge of Enterprise Management Strategy; knowledge of supply chain management, quality management, and international operation show a high load on the fourth factor, which is called knowledge of Enterprise Production and Operation. Therefore, the structure of the model for LBCC based on knowledge factors can be described by Figure 1.

### Establishment of the Model for LBCC Based on Skill Factors

In terms of skill, 19 items in two major categories (basic and managerial skills) were extracted from the scale of LBCC, as shown in Table 1. The KMO value reflecting the overall validity of skill factors in the questionnaires was 0.907, which implied that items related to various skill factors in the questionnaires contain many common factors. As a result, the validity through factor analysis is feasible. PCA was applied to conduct factor analysis and varimax rotation was used to implement orthogonal rotation on factor load matrix to thus obtain three factors. The interpretation rate of the factors for the total variance reached 63.274% and the result obtained through factor analysis is displayed in Table 7.

**Table 7:** Result of factor analysis on skill factors.

	Component		
	
	1	2	3
Position competency	0.751	0.073	0.153
Self-discipline ability	0.744	0.124	0.009
Executive capacity	0.724	0.168	0.334
Learning ability	0.640	0.295	0.325
Team-based organization ability	0.638	0.356	0.173
Stress resistance	0.598	0.319	0.274
Ability to understand policy	0.597	0.503	0.108
Comprehensive thinking ability	0.567	0.452	0.301
Flexible application ability	0.488	0.362	0.478
Resource integration capacity	0.157	0.780	0.183
Cross-cultural management ability	0.131	0.758	0.116
Ability of crisis management	0.200	0.755	0.246
Risk management capacity	0.342	0.731	0.081
Strategic decision-making ability	0.258	0.657	0.363
Innovation capacity	0.217	0.598	0.232
Communication ability	0.291	0.176	0.840
Ability of interpersonal skill	0.249	0.151	0.820
Negotiation ability	-0.016	0.359	0.777
Ability of analysis and identification	0.456	0.200	0.643


*Extraction method, principal component analysis.*

**FIGURE 2:**
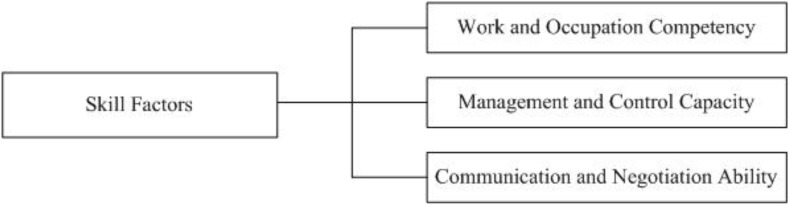
LBCC model based on skill factors.

According to the aforementioned factor analysis, three factors can be extracted from 19 skill factors. Therein, position competency, self-discipline ability, executive capacity, learning ability, team-based organization ability, stress resistance, ability to understand policy, comprehensive thinking ability, and flexible application ability show a high load on the first factor, which are called Work and Occupation Competency; resource integration capacity, cross-cultural management ability, ability of crisis management, risk management capacity, strategic decision-making ability, and innovation capacity exhibit a high load on the second factor, which are called Management and Control Capacity; communication ability, ability of interpersonal skill, negotiation ability, and ability of analysis and identification have a high load on the third factor, which are called Communication and Negotiation Ability. Therefore, the structure of the model for LBCC based on skill factors can be displayed in Figure 2.

### Establishment of the Model for LBCC Based on Attitude Factors

In terms of attitudes, 16 items were extracted from the scale for LBCC, as shown in Table 1. The KMO value reflecting the overall validity of attitude factors in the questionnaires was 0.917. This indicated that various attitude factors in the questionnaires have many common factors and therefore the validity through factor analysis is feasible. Factor analysis was carried out using PCA and the factor load matrix was subjected to orthogonal rotation by using varimax rotation to thus attain two factors. The interpretation rate of the factors for the total variance was 63.642% and the result obtained based on factor analysis is shown in Table 8.

**Table 8:** Result of factor analysis on attitude factors.

	Component	
	
	1	2
Team awareness	0.789	0.260
Win-win cooperation	0.773	0.264
Tenacity and determination	0.772	0.355
Objectivity and fairness	0.735	0.365
Achievement motive	0.725	0.074
Aggressiveness	0.713	0.400
Honesty and trustworthiness	0.708	0.398
Dialectical thinking	0.590	0.459
Rationality and calmness	0.569	0.529
Caution	0.497	0.473
Legal consciousness	0.208	0.867
Rule consciousness	0.245	0.827
Sense of responsibility	0.458	0.696
Carefulness	0.480	0.637
Consciousness of rights safeguarding	0.152	0.606
Overall viewpoint	0.568	0.599


*Extraction method, principal component analysis.*

**FIGURE 3:**
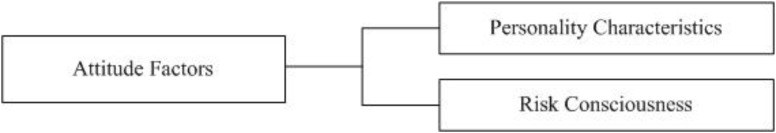
LBCC model based on attitude factors.

According to the aforementioned factor analysis, two factors can be extracted from 16 attitude factors. Among them, team awareness, win-win cooperation, tenacity and determination, objectivity and fairness, achievement motive, aggressiveness, honesty and trustworthiness, dialectical thinking, rationality, calmness, and caution show a high load on the first factor, which can be called Personality Characteristics; legal consciousness, rule consciousness, sense of responsibility, carefulness, consciousness of rights safeguarding, and overall viewpoint exhibit a high load on the second factor, which can be called Risk Consciousness. Thus, the structure of the model for LBCC based on attitude factors can be described in Figure 3.

### The Influence of Various Factors on LBCC

Through factor analysis, it can be seen that LBCC can be well explained by using nine factors, involving knowledge of standard of enterprise operation, knowledge of enterprise asset management, knowledge of enterprise management strategy, knowledge of enterprise production and management, work competency, management and control capacity, communication and negotiation ability, personality characteristics, and risk consciousness. The nine factors are used to conduct multiple regressions on LBCC to analyze the influence of various factors on the LBCC. Assuming that *Y* refers to LBCC and the aforementioned nine factors are separately represented by applying FAC1 to FAC9. The model is as follows:

$$\begin{array}{ll} Y_{i} & =b0+b1\mathrm{FAC}1+b2\mathrm{FAC}2+b3\mathrm{FAC}3+b4\mathrm{FAC}4+b5\mathrm{FAC}5\\ & +b6\mathrm{FAC}6+b7\mathrm{FAC}7+b8\mathrm{FAC}8+b9\mathrm{FAC}9+\xi i \end{array}$$

By using SPSS, multiple regressions are carried out on the model and the result of data analysis is shown in Table 9. It can be seen from the regression coefficient of the model that, when regression equation contains the above nine variables, the significance probability of the equation is lower than 0.001, rejecting the null hypothesis that the population regression coefficients are all equal to zero, therefore, the model should contain the nine variables. It can be seen from the analysis of regression coefficients that the significance level of regression coefficients is lower than 0.05, which is verified by *t*-test. That is, the standardized multiple regression model is as follows:

$$\begin{array}{ll} Y & =3.635+0.302\mathrm{FAC}1+0.266\mathrm{FAC}2+0.246\mathrm{FAC}3+0.213\mathrm{FAC}4+0.227\mathrm{FAC}5\\ & +0.228\mathrm{FAC}6+0.18\mathrm{FAC}7+0.233\mathrm{FAC}8+0.21\mathrm{FAC}9 \end{array}$$

**Table 9:** Coefficients of regression equation for LBCC.

		Non-standardized coefficient		Standardized coefficient	t	Sig.
				
		*B*	Standard error	Beta		
	(Constant)	3.635	0.001		4576.263	0.000
Knowledge factors	FAC1: knowledge of standard of enterprise operation	0.124	0.001	0.302	134.878	0.000
	FAC2: knowledge of enterprise asset management	0.110	0.001	0.266	129.291	0.000
	FAC3: knowledge of enterprise management strategy	0.101	0.001	0.246	102.931	0.000
	FAC4: knowledge of enterprise production and management	0.087	0.001	0.213	102.602	0.000
Skill factors	FAC5: work competency	0.094	0.001	0.227	76.027	0.000
	FAC6: management and control capacity	0.094	0.001	0.228	91.029	0.000
	FAC7: communication and negotiation ability	0.074	0.001	0.180	77.859	0.000
Attitude factors	FAC8: personality characteristics	0.096	0.001	0.233	83.166	0.000
	FAC9: risk consciousness	0.086	0.001	0.210	80.285	0.000


All factors are positively proportional to LBCC, implying that the improvement of abilities in the nine aspects can increase the LBCC to differing extents.

### The Influence of LBIEE on Graduates

The purpose of EE is to improve students’ comprehensive ability to adapt to complex environments in business activities (Rauch and Hulsink, 2015; Daniela et al., 2016). It is feasible to measure the training effect of EE based on two dimensions: objective compensation level and subjective job satisfaction of graduates in business activities.

Business education can bring long-term economic value for students, which significantly increases graduates’ compensation level (Jonathan et al., 2010). To observe the influence of LBIEE on the compensation level of graduates, the competency level of graduates in legal-business after receiving LBIEE was taken as the independent variable. Moreover, the annual salary of graduates was considered as a dependent variable. Through regression analysis, it can be seen, from Table 10, that the competency level in legal-business shows a significantly positive correlation with graduate salary. This indicates that LBIEE can improve the compensation level of graduates and the LBCC strengthens the comprehensive quality of graduates in career development. Graduates make a high-quality human capital investment in LBIEE.

**Table 10:** Data on the influence of LBIEE on compensation level of graduates.

Model		Non-standardized coefficient		Standardized coefficient	*t*	Sig.
				
		*B*	Standard error	Beta		
1	(Constant)	0.457	0.818		0.559	0.577
	LBCC	0.713	0.224	0.258	3.188	0.002


The level of job satisfaction of graduates is an overall view after comprehensively considering various factors and it is a key factor for measuring education quality (Yue, 2013). Education can indirectly influence job satisfaction of graduates through various factors including salary and physical conditions (Eugenia and Luis, 2007). Entrepreneurial activity not only can bring material welfare but enhance job satisfaction and well-being (Wim et al., 2014). To explore the influence of LBIEE on job satisfaction of graduates, the competency of graduates in legal-business contexts after receiving LBIEE was regarded as the independent variable. Moreover, the job satisfaction of graduates in business activities was considered as a dependent variable. Through regression analysis (Table 11), it can be seen that the competency level in legal-business contexts is positively proportional to graduate job satisfaction. This implied that LBIEE can improve graduate job satisfaction. The LBCC is not only conducive to improving the ability of graduates in commercial management context but can also strengthen their adaptability to complex external business competition during the transition of China’s economy. Establishing the concept of rule of law and rule consciousness is conducive to commercial development.

**Table 11:** The influence of LBIEE on graduate job satisfaction.

		Non-standardized coefficient		Standardized coefficient	*t*	Sig.
				
		*B*	Standard error	Beta		
1	(Constant)	48.727	9.134		5.335	0.000
	LBCC	6.759	2.497	0.221	2.707	0.008


## Conclusion and Discussion

The China University of Political Science and Law proposed the cultivation of LBITs characterized by possessing business and law intelligence, quick thinking, and strong actions. The university first launched the LBIEE project in 2010 and started an upsurge in innovative LBIEE modal development. LBIEE complies with the direction of innovating talent cultivation through EE in China’s universities and also satisfies the practical needs of standardized, legalized development of the market economy for cultivating interdisciplinary talents (Chai and Sun, 2012). The core task of LBIEE is to improve students’ LBCC and enable students to show entrepreneurial spirit with legal-business intelligence (Sun, 2012). For LBIEE, it needs to pay attention to cultivate legal-business intelligence in the following ways:

### Integrating Legal Thinking Into Business Logics

As an independent discipline, business EE has its complete course system, including core curriculum and specialized curriculum. The purpose of the design logic of courses is to cultivate leaders in commercial circles who exhibit relatively comprehensive knowledge systems in management and also show entrepreneurial spirit. Such an education program is likely to lead to homogenization (Stewart and Anne, 2003; Datar et al., 2013). LBIEE incorporates legal thinking into the system of business. In terms of curriculum provision, law courses related to business need to be set, for example, contract law, economic law, tax law, and WTO legal regulation (Wang, 2018). This can equip graduates with entrepreneurship-related laws and regulations and a knowledge bank of policy rules and a grasp of various skills (planning, marketing, decision-making, and risk assessment) required in entrepreneurship. The LBCC can effectively improve students’ entrepreneurial ability and urge them to take delight, and dare to participate, in entrepreneurship (Lu, 2015).

### Building Legal Awareness in Commercial Spirit

Traditional EE tends to emphasize innovation, breakthrough, and entrepreneurial spirit that dare to face failure. Many people think that it contradicts legal consciousness, and especially in the initial development period of China’s market economy, many excellent entrepreneurs have failed due to a lack of legal consciousness (Qu, 2015). LBIEE pays much attention to building correct values in business for students. By holding a series of courses, lectures, and case discussions related to legal-business, students’ commercial spirit is endowed with more connotative meaning. In this way, legal consciousness and business ethics imperceptibly influence students’ decision-making behaviors. The case-based course of most characteristic influence is law–business management, which realizes the integration of law and business knowledge in teaching research of management cases (Mu, 2012). On this basis, it is expected to train students to have an awareness of accepting laws and rules as a necessary constraint when facing practical problems in business.

### Legal Risk Prevention in Business Competition

Significant business competition implies significant risk: at present, during the transition of China’s economy, legal regulations have not yet been fully embedded and there are many uncertainties in market environment. Therefore, many enterprises frequently consider business risk during competition while ignoring legal risk. As a result, legal risk has been an important factor leading to entrepreneurship failure (Xie, 2017), which brings many unnecessary hidden dangers in business management for enterprise development (Baron et al., 2015). Additionally, Chinese enterprises are frequently subject to foreign law while implementing the “Going global” internationalization strategy. Transnational management cases evincing significant loss caused by China’s enterprises failing to evaluate legal risk have become common in recent years (Wang, 2012). LBIEE neither means cultivating entrepreneurs into professional legal talents nor requires students to learn legal provisions by rote in this training program: it lets students know of the legal issues and pitfalls in business management through law–business interdisciplinary course teaching and case discussion. Furthermore, entrepreneurs can achieve success in business by taking effective strategies to prevent legal risk.

### Strengthening the Application of Legal Personnel in Commercial Activities

Various management activities of an enterprise are always related to legal affairs. A majority of students who received law–business EE are professional managers in various domains at different positions. Besides, a batch of advanced professional layers in major law firms in China also participates in LBIEE and they bring their rich experience in legal affairs from their client portfolios. LBIEE leads to an interpersonal network satisfaction for sharing legal-business experience (Sun, 2012). Through interaction between students, practical problems of students in enterprises are discussed as law–business cases (Ge and Mu, 2010), which provide systematic solutions to business activities by using law–business conjoined thinking while also reflecting the importance of participating in LBIEE.

### Limitations and Suggestions

This research is an exploratory factor analysis for establishing the model of LBCC by the process and methods of competency research. However, due to the limited purpose of this study, the training effect of the LBIEE project is only examined by the influence on objective compensation level and subjective job satisfaction of the graduates. Empirical studies relating to the effects of LBIEE projects on other aspects of graduates (e.g., entrepreneurial intention) need to be added and strengthened in the future researches for better improving this pioneering EE mode. In addition, the survey of this research only focus on Business School of China University of Political Science and Law (CUPL) started to explore the innovation of the LBIEE mode, which limits the generalizability of the results. Future researches ought to expand the samples to study individuals in different LBIEE projects. Moreover, this research is primarily based on the Chinese context because the LBIEE mode adapted to characteristics of China’s market transition which requires a great number of LBITs who not only have a command of management but also show legal awareness. Whether the LBIEE mode is suitable for the development environment of foreign countries has not been discussed. The wider applicability of this LBIEE mode can be further explored in future researches.

## Author Contributions

TW provided substantial contributions to the research design and data analysis. TW wrote and revised the manuscript. TW approved of this version of the manuscript to be published.

## Conflict of Interest Statement

The author declares that the research was conducted in the absence of any commercial or financial relationships that could be construed as a potential conflict of interest.
